# Seroprevalence of *Toxoplasma gondii* in wild boars (*Sus scrofa*) from Central Italy

**DOI:** 10.1051/parasite/2013048

**Published:** 2013-11-28

**Authors:** David Ranucci, Fabrizia Veronesi, Annabella Moretti, Raffaella Branciari, Dino Miraglia, Maria Teresa Manfredi, Daniela Piergili Fioretti

**Affiliations:** 1 Department of Biopathological Science and Hygiene of Animal Production and Food, University of Perugia, Via San Costanzo 4 06126 Perugia Italy; 2 Department of Veterinary Sciences and Public Health, University of Milano, Via Festa del Perdono 7 20122 Milano Italy

**Keywords:** Wild boar, *Toxoplasma gondii*, Seroprevalence, Italy

## Abstract

Wild and farmed game meat consumption has been highlighted as an emerging risk factor for *Toxoplasma gondii* infection in humans. In Central Italy wild boar is widely distributed and is also one of the most popular game species. The main goal of the present study was to estimate the seroprevalence of *T. gondii* antibodies through a serological survey conducted on 400 hunted wild boars (250 males and 150 females) during three subsequent hunting seasons (2009–2011), using an Immunofluorescence Antibody Assay. The animals were sorted by age, determined on the evaluation of the dental table; 101 were <1 year old, 175 from 1 to 3 years, and 124 > 3 years. Antibodies against *T. gondii* were detected in 56 (14%) serum samples with titers ranging from 40 to ≥160; a significant association (*p* < 0.05) was found between seropositivity and age, but not gender, hunting districts, or year of sampling.

## Abstract (fr)

Il a été souligné que la consommation de viande de gibier sauvage et d’élevage est un facteur de risque émergent pour l’infection toxoplasmique humaine. En Italie centrale le sanglier est largement distribué et est également l’une des espèces de gibier les plus populaires. L’objectif principal de la présente étude était d’estimer la séroprévalence des anticorps contre *Toxoplasma gondii* par une enquête sérologique réalisée sur 400 sangliers chassés (250 mâles et 150 femelles) pendant 3 saisons de chasse consécutives (2009–2011), en utilisant un test d’immunofluorescence. Les animaux ont été classés par âge, déterminé sur l’évaluation de la table dentaire; 101 étaient âgés de <1 an, 175 de 1 à 3 ans et 124 > 3 ans. Les anticorps contre *T. gondii* ont été détectés dans 56 (14 %) des échantillons de sérum avec des titres allant de 40 à ≥ 160 ; une association significative (*p* < 0,05) a été trouvée entre la séropositivité et l’âge, mais pas avec le genre, les districts de chasse, ou de l’année de l’échantillonnage.

## Introduction

*Toxoplasma gondii* is an important tissue cyst-forming coccidia distributed worldwide, which infects several species of homeothermic animals including humans, with medical and veterinary consequences [1]. Human toxoplasmosis is the most common parasitic zoonosis in the European Union (0.56 cases out of 100,000 inhabitants) [11]. In recent years wild and farmed game meat consumption has started to be considered an emerging risk factor for *T. gondii* infection in humans [13], and European legislation [8] now includes this pathogen in the list of zoonotic agents to be subject to epidemiological monitoring, in wild animals too. The most recent estimates provided by the European Food Safety Authority [10] reported that approximately 50% of wild game is seropositive for *T. gondii.* However information on the recent prevalence of infection in hunted wild boar (*Sus scrofa*, Linnaeus, 1758), one of the most popular large game animal species in Europe, is limited [9, 15, 16, 20] and almost non-existent for Italy [6, 14, 17]. In the region of Umbria, Central Italy, wild boar is widely distributed and is the most popular large game species. A marked increase in the population density of wild boars has been observed in the last 40 years, reaching peaks of maximum spread in the north-east of the region where hunting has become very intensive [18]. The hunted animals are usually directly consumed by hunters and their families, or utilized to produce products supplied to the local market. Since wild boar meat has been demonstrated to be a potential source of human infection [4], a careful evaluation of the prevalence of *T. gondii* infection in hunted animals is needed to protect public health. The purpose of the present study was to survey the prevalence of *T. gondii* infections in hunted wild boar in Umbria, Central Italy.

## Materials and methods

Four hundred wild boars (250 males and 150 females) shot in the Central-Northern areas of Umbria during three subsequent hunting seasons (2009–2011), in line with the reduction plan in progress, were included in the epidemiological survey. The wild boars originated from four nearby hunting districts (Hd) in the region (Hd 3: from 43°31′59,66″N to 43°23′37,47″N and from 12°11′10,69″E to 12°24′48,16″E; Hd 8: from 43°26′49,47″N to 43°15′13,87″N and from 12°12′6,52″E to 12°29′55,72″E; Hd 9: from 43°17′10,05″N to 43°6′48,81″N and from 12°14′54,04″E to 12°30′37,48″E; Hd 10: from 43°17′0,50″N to 43°1′55,88″N and from 12°1′47,90″E to 12°17′4,53″E; http://www.atcperugia1.it/cartografieearth.html); in each of these hunting districts 100 animals were sampled over the 3 years considered. The animals were sorted by age, determined on the evaluation of the dental table; 101 were <1 year old, 175 from 1 to 3 years, and 124 > 3 years. Blood samples were collected directly by hunters from each animal by cardiac punctures, placed into sterile tubes without anticoagulant and centrifuged (4.000 rpm for 15 min). The sera obtained were collected and stored at −20 °C until the time of analysis. All serum samples were screened by Immunofluorescent Antibody Test (IFAT) (Diagnostik Megacore, Horbranz, Austria) for the detection of anti-*T. gondii* specific IgG, as described by Ranucci et al. [19]. Sera were tested at a screening dilution of 1:40 (cut off), a titer validated to detect IgG antibodies to *T. gondii* in sera of wild boar [2]; the positive samples were subjected to twofold serial dilutions to determine the final titer (end-point). Sera of *T. gondii*-free and naturally infected wild boar were included in each reaction as positive and negative controls.

The overall seroprevalence for *T. gondii* was calculated and the hypothesized risk factors (gender, age, hunting districts, and sampling year) were individually screened for association with the likelihood of *Toxoplasma* seropositivity. Chi-squared analysis was used for this purpose; the results for each variable were expressed as *p* value and, when significant, odds ratio (OR) with a 95% confidence interval (CI) were calculated. All statistical analyses were performed using the WINPEPI (PEPI-for-Windows) freeware epidemiological software with the *p* value set at 0.05.

## Results

Fifty-six out of the 400 (14%, 95% CI: 9.9–18.1%) serum samples examined were found to be positive for *T. gondii* antibodies with titers ranging from 40 to ≥160; 32 sera exhibited a titer of 40, 18 of 80 and 6 a titer ≥160.

The results of the univariate analysis are shown in Table 1. A significant association (*p* < 0.05) was found between age and the presence of *T. gondii* antibodies: animals > 3 years of age showed the highest seroprevalences (19.3%, 95% CI: 15.69–22.91%) with an OR of 3.8 (95% CI: 1.2–4.3) followed by wild boars aged between 1 and 3 (OR: 2.7; 95% CI: 2.5–6.3). The antibodies reactive to *T. gondii* was not significantly associated with gender, nor with the hunting districts where the sampled wild boars originated nor the sampling year (*p* > 0.05).

**Table 1.** Risk factors associated with *Toxoplasma gondii* seropositivity in hunted wild boar.

Variables	No. positive animals/animal tested (%)	*p*-value
*Gender*		
male	34/250 (13.6%)	0.11
female	22/150 (14.7%)	
*Age*		
< 1 year	6/101 (0.6%)	
1–3 years	26/175 (14.8%)	0.01
> 3 years	24/124 (19.3%)	
*Hunting district*		
3	12/100 (12%)	
8	15/100 (15%)	
9	17/100 (17%)	0.70
10	12/100 (12%)	
*Sampling year*		
2009	19/134 (14.2%)	
2010	23/163 (14.1%)	0.99
2011	14/103 (13.6%)	

## Discussion and conclusions

*T. gondii* infection is considered to be prevalent in wild boar populations worldwide [3]. The wild pig can be exposed to toxoplasmosis through contact with food or water contaminated with sporulated oocysts from felid species and/or by consuming infected tissues of intermediate hosts, due to its foraging among fox and rodent carcasses [3]. Wild boar play an important role in maintaining a sylvatic cycle of the parasite (e.g., wild boars–foxes– rodents–wild birds) [2, 3], favored by bad practice among hunters, who leave residues of wild boar carcasses in the fields which can be scavenged by other wild animals [12]. For all the aforementioned reasons wild boars appear to be ideal indicators for understanding geographical variations associated with the prevalence of toxoplasmosis in the wild [2]. The prevalence rate of *T. gondii* infection detected in the present survey (14%) shows a moderately wide spread of the parasite among the wild pig population investigated, lower than that found in Northern Italy [15], but very similar to that found in Umbria in a previous epidemiological survey conducted on 960 domestic finishing pigs (prevalence rate 16.14%) [22]. This result is not in accordance with the observation of Opsteegh [15] who found that seroprevalence in wild boars is much lower than in Dutch fattening pigs (24.4% *versus* 60%).

Recent surveys on the prevalence of *T. gondii* in wild boar throughout Europe showed wide variations in serum ranging from 8.1% to 38.4% [9, 15, 16, 20]. However an overall comparison of the prevalence observed with data reported in other countries is difficult and should be performed with caution due to the different conditions and techniques of each study. There are geographical variations among the different study areas, different sampling procedures, animal populations (i.e. free-living, or reared animals), and there is a lack of standardization of the diagnostic techniques (varying sensitivity, specificity, cut-off values) [9].

As regards the risk factors considered, the results of the univariate analysis showed that gender, hunting district and year of sampling made no significant difference to *T. gondii* seroprevalence, but did show that age is a significant risk factor. These results are in agreement with data obtained by Antolová et al. [1] showing a significantly higher seroprevalence in adult wild boar than in young boar [1]. They also concur with the results obtained by Opsteegh et al. [15] showing an absence of a significant effect of temporal or regional variations on seroprevalence, which could indicate a stable and homogeneous infection pressure from the environment. Several Authors, however, did not observe a statistically significant effect of age on seroprevalence of *T. gondii* in wild boar [7, 21], and Opsteegh et al. [15] found that the mean age of the animals showed a step-increase in seroprevalence up to 10 months, but a stable situation thereafter. These observations seem to be inconsistent with a lifelong persistence of immunity against *T. gondii* in wild boars. However before drawing this conclusion, longitudinal studies on the follow up of antibodies in the infected animals are required.

In conclusion the results obtained in the present survey indicate that a circulation of *T. gondii* exists in the wild boar populations of Central Italy. A reliable risk assessment of human toxoplasmosis caused by the consumption of wild boar meat products is not currently available. Even though the risk has been commonly considered of scant importance in countries such as Italy due to the traditional careful cooking of game meat, nonetheless we have to consider that old culinary habits are being replaced with new trends and consumers nowadays may eat inadequately cooked or raw meat and meat products. The importance of wild boars as a source of human infection could therefore be on the increase [5]. Moreover the hunters’ practice of leaving residues of wild boar carcasses in the environment, and the handling of carcasses, could result in direct human infection [4], transmission of the parasite and an increase of the potential risk of human infection. For this reason it is advisable to insist that hunters, like other staff handling foodstuffs, undergo training on health risks.

Further studies focusing on the isolation of viable parasites from tissues of seropositive wild boars and on the genetic characterization by PCR-RFLP and multilocus microsatellite analysis of the different strains/genotypes distributed among the wild boar populations are needed to clarify the real zoonotic risk for humans.
